# Ten‐Eleven Translocation Enzymes Control the Rate and Mode of Retinal Progenitor Cell Division in the Developing Retina

**DOI:** 10.1096/fj.202603335R

**Published:** 2026-09-25

**Authors:** Galina Dvoriantchikova, Michelle Fleishaker, Byron L. Lam, Dmitry Ivanov

**Affiliations:** ^1^ Department of Ophthalmology, Bascom Palmer Eye Institute University of Miami Miller School of Medicine Miami Florida USA; ^2^ Department of Ophthalmology, Illinois Eye and Ear Infirmary University of Illinois College of Medicine Chicago Illinois USA; ^3^ Department of Microbiology and Immunology University of Miami Miller School of Medicine Miami Florida USA

**Keywords:** cell cycle, DNA demethylation, epigenetics, photoreceptors, retinal development, retinal progenitor cells, TET enzymes

## Abstract

Ten‐Eleven Translocation (TET) enzymes are responsible for DNA demethylation and epigenetic regulation. Inactivation of TET enzymes in retinal progenitor cells (RPCs) leads to the emergence of a non‐functional retina, resulting in blindness. However, it was not known how the inactivation of TET enzymes in RPCs affects their immediate function. To this end, we investigated TET‐deficient RPCs in the developing retina using various approaches including EdU cell proliferation assay, immunohistochemistry, bulk RNA‐seq, and snRNA‐seq. We found that inactivation of TET enzymes in RPCs results in slow retinal growth. The number of dividing TET‐deficient RPCs in these retinas is lower, whereas the number of non‐dividing cells derived from them is significantly higher compared to controls. This phenomenon could be explained by the predominantly asymmetric division of TET‐deficient RPCs, which is characteristic of late progenitor cells. At the same time, TET‐deficient RPCs maintained a high rate of proliferation, continuing to divide even during late stages of retinal development, a phase during which cell division could no longer be detected in the control samples. A high rate of proliferation is a characteristic typically associated with early progenitor cells. We showed that retinas emerging from the activity of these TET‐deficient RPCs are less developed and less complex, likely due to the mixed phenotype of the RPCs. Thus, our findings suggest that the activity of TET enzymes in RPCs is necessary to prevent mixing of their early and late phenotypes, a condition that is critical for normal retinal development.

## Graphical abstract

Early RPCs divide predominantly symmetrically and exhibit a high proliferation rate, resulting in rapid retinal growth. Late RPCs divide predominantly asymmetrically and exhibit a low proliferation rate. Our results suggest that TET‐deficient RPCs divide predominantly asymmetrically but exhibit a high proliferation rate. This results in slow retinal growth yet ensures the presence of dividing cells at the latest stage of retinal development. Alterations in the cellular composition of TET‐deficient retinas are likely caused by the mixed phenotype of their RPCs.

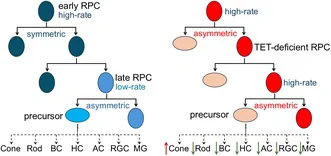

## 1 Introduction

The retina consists of several types of neurons whose function is to convert light into electrical signals (rod and cone photoreceptors) and then transmit them to the brain (bipolar, horizontal, amacrine, and ganglion cells) to create the sensation of vision [1, 2, 3, 4]. Impaired development of these neurons can significantly affect retinal function and even lead to blindness [5]. All retinal neurons and Müller glia differentiate from retinal progenitor cells (RPCs), which, like all progenitors, are classified into early and late types based on their properties. Early RPCs differentiate into early‐born neurons (cones, ganglion, horizontal cells, and a subpopulation of amacrine cells) and late RPCs [1, 2, 3, 4]. Late RPCs differentiate into late‐born neurons (rods, bipolar cells, and a subpopulation of amacrine cells) and Müller glia [1, 2, 3, 4]. Early RPCs exhibit a high rate of proliferation and predominantly symmetric self‐renewal [3, 6, 7, 8]. This leads to an exponential increase in cell number and rapid retina growth. Late RPCs divide slowly and predominantly asymmetrically, generating one progenitor and one non‐dividing precursor of a certain cell type [3, 6, 7, 8]. This results in the emergence of a variety of retinal cell types. The timely activity of early and late RPCs is a crucial condition for the normal development of the retina.

The development of a tissue is accompanied by changes in the patterns of methylated cytosines in the DNA of its constituent cell types [9, 10, 11]. These methylation patterns maintain optimal patterns of gene expression necessary for normal cell activity in tissue [9, 10, 11]. There are two families of enzymes that are responsible for the formation of DNA methylation patterns in cells: enzymes belonging to the DNA methyltransferase (DNMT) family catalyze the transfer of a methyl group to the cytosine nucleotide, while the Ten‐Eleven Translocation (TET) family promotes DNA demethylation [9, 10, 11]. Changes in DNA methylation patterns are critical to retinal development since many genes essential for the development and function of the various retinal cell types are hypermethylated in RPCs from which these cell types originate [12, 13, 14, 15, 16]. Active demethylation of these genes is carried out by TET enzymes, which explains their crucial role in the development of the retina: genetic ablation of the TET gene family in RPCs impairs retinal development and leads to blindness [17, 18, 19]. Given the crucial role of TET enzymes in retinal development, we know virtually nothing about how the inactivation of these enzymes in RPCs directly affects RPC function.

To address the knowledge gaps, we studied the development of TET‐deficient and control retinas from the embryonic stage using EdU cell proliferation assay, snRNA‐seq, and bulk‐RNA‐seq. We found that TET‐deficient RPCs exhibit characteristics of both early and late progenitors, a factor that most likely contributes to the disrupted cellular composition of the retina, wherein the cone population expands significantly at the expense of all other cell types. The differentiation of TET‐deficient RPCs also results in the formation of populations of abnormal retinal cell types, a phenomenon particularly evident in the development of rod and cone photoreceptors. Taken together, these results point to a mechanism by which TET enzymes control RPC function in the developing retina.

## 2 Materials and Methods

### 2.1 Ethics Statement

The procedures were performed in compliance with the NIH Guide for the Care and Use of Laboratory Animals, ARVO statement for the Use of Animals in Ophthalmic and Vision Research, ARRIVE (Animal Research: Reporting of In Vivo Experiments) guidelines, and according to the University of Miami Institutional Animal Care and Use Committee (IACUC) approved protocol #: 23‐058. Animals were euthanized according to the recommendations of the Panel on Euthanasia of the AVMA.

### 2.2 Animals

Tet1‐floxed, Tet2‐floxed, and Tet3‐floxed mice were obtained from Dr. Anjana Rao (La Jolla Institute for Immunology) and were crossbred to produce TET triple floxed mice (they are designated in the text as TET). These animals were used as controls and also to generate TET conditional knockouts. To this end, Chx10‐Cre mice (strain 005105, the Jackson Laboratory, Bar Harbor, ME, USA) and TET triple floxed mice were crossbred to produce TET triple conditional knockout mice (they are designated in the text as Chx10TET). Chx10TET mice were heterozygous for the Chx10‐Cre allele and homozygous for Tet1‐floxed, Tet2‐floxed, and Tet3‐floxed alleles. Chx10TET and TET mice have the C57BL/6J genetic background. Embryonic retinas were collected from embryonic day 11 (E11) C57BL/6J mice (strain 000664, the Jackson Laboratory, Bar Harbor, ME, USA). Both females and males were used to address sex as a biological variable. The animals were kept in standard housing conditions on a 12‐h light/dark cycle with unrestricted access to food and water.

### 2.3 Tissue Collection

To collect retinas without contamination by blood cells, mice were perfused with phosphate buffered saline (PBS, pH 7.4; #10010023, ThermoFisher Scientific, Waltham, MA, USA). To this end, the mice were IP injected with ketamine (80 mg/kg) and xylazine (10 mg/kg). When the mice were under deep anesthesia, the chest cavity was opened, and a syringe needle was placed into the heart. The syringe needle was attached to a pump to allow phosphate‐buffered saline (PBS) to circulate through the mouse. Eyes were then dissected out and retinas were isolated. The retinas were collected in the appropriate buffers for RNA and DNA purification, and immunohistochemistry.

### 2.4 Immunohistochemistry

Enucleated eyes were fixed in 4% paraformaldehyde (PFA) overnight at 4°C. The fixed eyes were placed in 30% sucrose/PBS. After 24 h, the eyes were cryo‐embedded in OCT and cryo‐sectioned at 10 μm thickness. We then permeabilized the sections with 0.3% Triton X‐100/PBS for 1 h, washed with PBS, and blocked in a buffer containing 5% donkey serum, 2% BSA, and 0.15% Tween‐20 in PBS. After 1 h, primary antibodies and/or PNA (L32458, ThermoFisher Scientific, Waltham, MA, USA) in the blocking buffer were added to the sections. We used the following antibodies: anti‐Chx10 (Vsx2; 1:200, AB9016, MilliporeSigma, Burlington, MA, USA), anti‐ChAT (1:200, AB114P, MilliporeSigma, Burlington, MA, USA), anti‐Cplx4 (1:200, 122 402, Synaptic Systems GmbH, Goettingen, Germany), anti‐Cplx3 (1:500, 122 302, Synaptic Systems GmbH, Goettingen, Germany), anti‐Dlg4 (1:500, GTX133255, GeneTex, Irvine, CA, USA). The sections were incubated overnight at 4°C. The next day, the retinal sections were washed with PBS and incubated with secondary fluorescent antibodies (ThermoFisher Scientific, Waltham, MA, USA). Control sections were incubated without primary antibodies. Hoechst 33342 (H3570, ThermoFisher Scientific, Waltham, MA, USA) was applied to stain the cell nuclei. Images were collected and analyzed using the Leica STELLARIS confocal microscope (Leica Microsystems, Deerfield, IL, USA) and its software.

### 2.5 EdU Cell Proliferation Assay in Vivo

To label proliferating cells in the developing retina we used EdU‐Click kit (BCK‐EDU647, MilliporeSigma) according to the manufacturer's instructions. To this end, the EdU (50 mg/kg in 1X PBS) solution was injected intraperitoneally into pregnant mice (E16) or pups (P0–P10). After 4 h (pulse) or 30 min (short pulse), the retinas were collected and used in the immunohistochemistry analysis.

### 2.6 TUNEL Assay

To detect dead cells, we used the Click‐iT Plus TUNEL Assay Kit (C10617, ThermoFisher Scientific) according to the manufacturer's instructions.

### 2.7 Bulk RNA‐Seq Library Preparation and Sequencing

Total RNA was purified from retinas using RNeasy Plus Mini Kit (#74134, Qiagen, Hilden, Germany) [20]. The quantity and quality of RNA were assessed using the NanoDrop One Spectrophotometer and Qubit 4 Fluorometer. To assess RNA integrity, we used the 2100 Bioanalyzer Instrument (Agilent Technologies, Santa Clara, CA, USA). RNA samples with a RIN (RNA Integrity Number) score of 8 or higher were used to prepare RNA‐seq libraries. The mRNA‐seq library preparation Kit (RK20302, ABclonal, Woburn, MA, USA) was utilized according to the manufacturer's instructions to prepare RNA‐seq libraries, which were sequenced on the Illumina NovaSeq X Plus using 2 × 150 PE configuration. The FASTQ files have been deposited in the BioProject database (ncbi.nlm.nih.gov/bioproject/) and can be accessed using the accession number PRJNA1286813.

### 2.8 Bulk RNA‐Seq Data Analysis

We used the STAR RNA‐seq aligner, HTseq package, and GENCODE M25 (GRCm38.p6) *Mus musculus* reference genome (mm10) to compute how many reads overlap each of the mouse genes [21, 22]. The edgeR Bioconductor package was applied to perform the differential gene expression analysis [23]. We applied the DESeq2 Bioconductor package to perform the principal component analysis (PCA) and sample clustering [24].

### 2.9 Single‐Nucleus RNA Library Preparation, Sequencing and Data Analysis

Retinas were collected from P14 TET and Chx10TET mice and immediately frozen. Cell nuclei from the frozen retinas were isolated using the Nuclei Extraction Buffer (130‐128‐024, Miltenyi Biotec, Gaithersburg, MD, USA) and used to generate the 10X snRNA‐seq library according to the manufacturer's instruction (Chromium GEM‐X Single Cell 3′ Reagent Kits v4, 10× Genomics, Pleasanton, CA, USA). Briefly, the GEM generation and barcoding were done with a 10× Chromium X series. mRNA from the uniquely barcoded cell nuclei was subsequently reverse transcribed into cDNA, which was then amplified to generate sufficient amount for snRNA‐seq library construction. Next the cDNA was used for the bulk library preparation, including enzymatic fragmentation, size‐selection, end‐repair, A‐tailing, adaptor ligation and dual index PCR. Finally, the library was ready for quality control and followed sequencing on Illumina Novaseq X plus platform using PE150 strategy. The FASTQ files generated in the study have been deposited in the BioProject database (ncbi.nlm.nih.gov/bioproject/) and can be accessed using the accession number PRJNA1286813. To demultiplex next‐generation sequencing (NGS) data (FASTQ files), process barcodes, align reads to GRCm38.p6/mm10 (GENCODE M25 release) reference genome, and generate feature‐barcode matrices, we used the Cell Ranger software suite developed by 10× Genomics. The output from Cell Ranger was used in R toolkit Seurat (v5.2.0) for clustering, cell type annotation, differential expression analysis, and visualization. During quality control, only those cells that met the following conditions were retained: nCount_RNA > 500 and nFeature_RNA > 500 and mitoPercent < 5 and riboPercent < 5. In order to identify doublets and remove them we used the scDblFinder R Bioconductor package [25]. We used the SoupX R package to remove ambient RNA contamination from our snRNA‐seq data [26]. The R code that was used in snRNA‐seq analysis can be found in Data S1.

### 2.10 Statistical Analysis

The Student's *t*‐test was applied for experiments containing one variable. We considered *p*‐values and FDR equal to or less than 0.05 statistically significant. Generation and analysis of next‐generation sequencing data were performed in‐house according to ENCODE standards and pipelines. The relevant details can be found above.

## 3 Results

### 3.1 TET‐Deficient RPCs Predominantly Divide Asymmetrically

**FIGURE 1:**
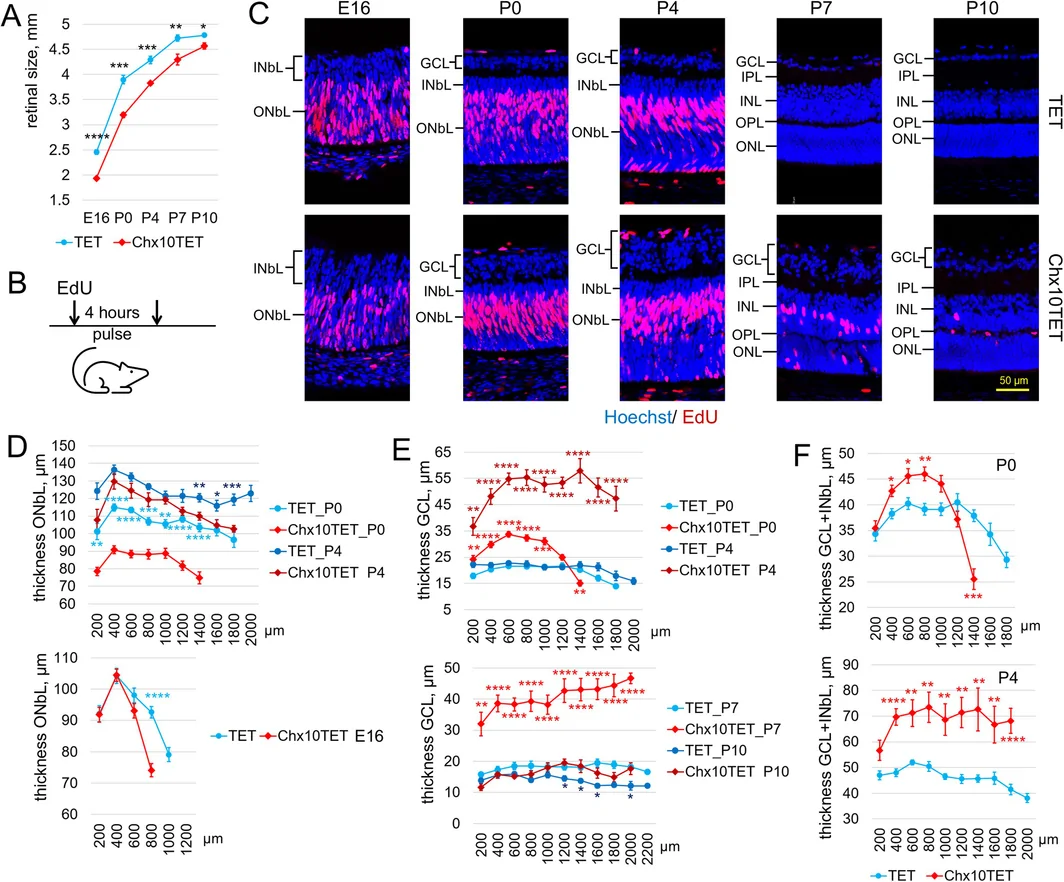
Predominantly asymmetric division of RPCs likely accounts for the phenotype of TET‐deficient developing retinas. (A) Genetic ablation of the TET family in RPCs affects the growth of the developing retina (*n* = 5 mice per group, per age; ****p < 0.0001, ***p < 0.001, **p < 0.01, *p < 0.05). (B) The EdU solution was injected intraperitoneally into mice. After 4 h (pulse), the retinas were collected and used for analysis. (C) Representative confocal images of developing TET‐deficient (Chx10TET) and control (TET) retinas treated with EdU (GCL, ganglion cell layer; INbL, inner neuroblastic layer; INL, inner nuclear layer; IPL, inner plexiform layer; ONbL, outer neuroblastic layer; ONL, outer nuclear layer; OPL, outer plexiform layer). (D–F) Measurements of ONbL (D), GCL (E), and GCL + INbL (F) thicknesses indicate that the number of dividing RPCs (ONbL) predominates in control (TET) retinas, whereas the number of non‐dividing precursors (GCL and GCL + INbL) predominates in TET‐deficient (Chx10TET) retinas (*n* ≥ 5 mice per group, per age). Statistical significance is shown between experimental and control retinas collected at the same time point.

The main functions of RPCs are to divide to allow the retina to grow and then differentiate into various cell types. To investigate the impact of TET enzymes on RPC function, we genetically inactivated the TET gene family in RPCs. To this end, we crossed triple Tet1/Tet2/Tet3‐floxed mice (TET) with Chx10‐Cre mice that express Cre recombinase in RPCs. We used the resulting TET‐deficient (Chx10TET) mice in our experiments (TET mice served as controls). By analyzing the size of experimental and control developing retinas collected from embryonic day 16 (E16), postnatal day 0 (P0), P4, P7, and P10 mice, we found that Chx10TET retinas were smaller than TET retinas at all time points examined (Figure 1A). The smaller size could result from either a low rate of RPC proliferation or a predominance of asymmetric division (one RPC and one non‐dividing retinal precursor). In the latter scenario, retinal growth proceeds more linearly, contrasting with the exponential growth associated with symmetric RPC division (two RPCs). Consequently, a predominance of asymmetric division would lead to a lower number of RPCs and a higher number of non‐dividing cells in Chx10TET versus TET retinas. To test this hypothesis, we labeled dividing RPCs using the EdU assay (Figure 1B). This allowed us to identify the outer neuroblastic layer (ONbL) containing RPCs, as well as the inner neuroblastic layer (INbL) and the ganglion cell layer (GCL), which contain non‐dividing cells (Figure 1C). We measured the thickness of these layers, which reflects the number of cells in them. We found that the thickness of the ONbL was smaller in Chx10TET versus TET retinas (Figure 1D, Figure S1). Conversely, the GCL was abnormally thick in TET‐deficient retinas (Figure 1C,E, Figure S1). The total combined thickness of the GCL and INbL was also greater in Chx10TET versus TET retinas (Figure 1F). Our findings indicate that the number of dividing RPCs is lower and the number of non‐dividing cells is higher in developing Chx10TET versus TET retinas, which supports our hypothesis regarding the predominantly asymmetric division of TET‐deficient RPCs.

### 3.2 TET‐Deficient RPCs Exhibit a High Rate of Proliferation

**FIGURE 2:**
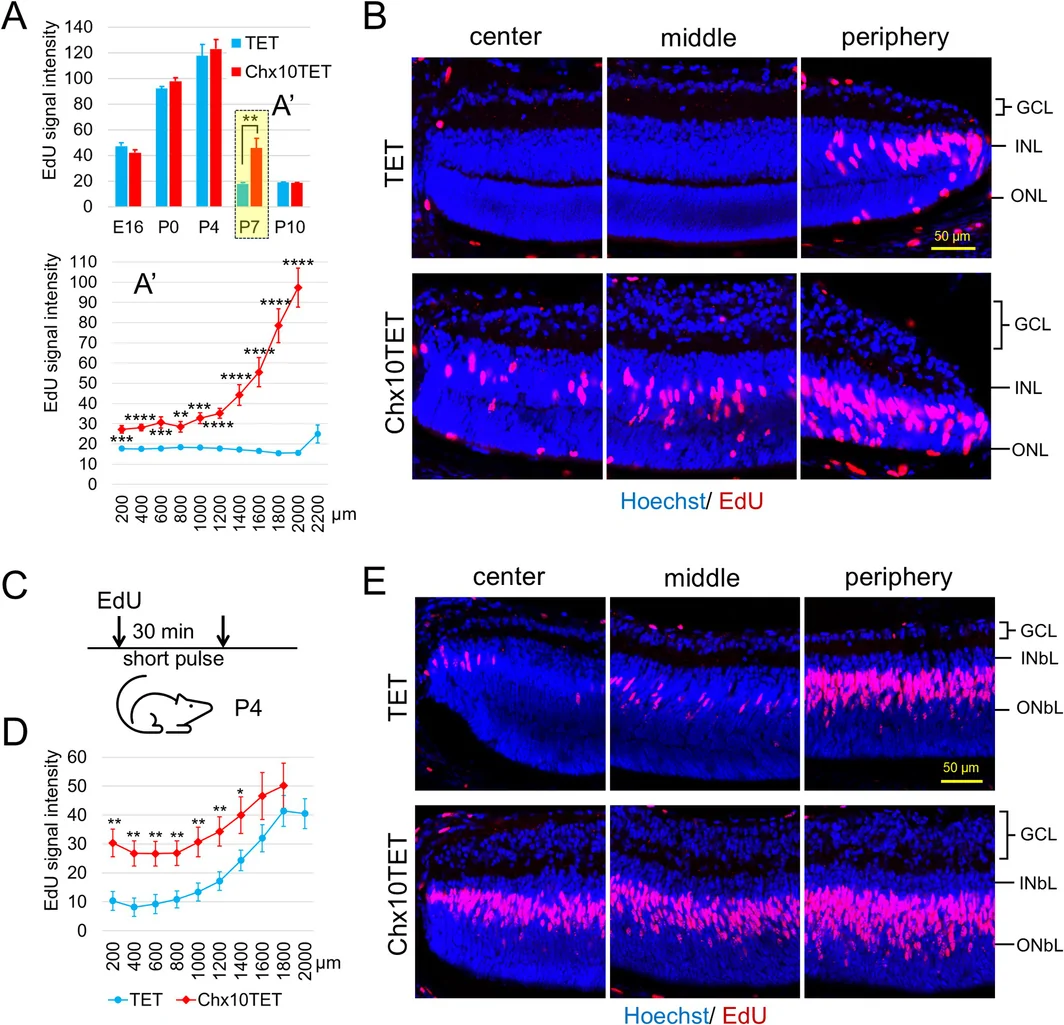
TET‐deficient RPCs continue to divide even when control RPCs have already stopped. (A) The results of measurements of mean fluorescent EdU signal intensity (A, A') revealed an anomaly in the number of dividing TET‐deficient RPCs at P7 (*n* ≥ 5 mice per group, per age; **p < 0.01, ***p < 0.001, ****p < 0.0001). (B) The panel shows representative confocal images of retinal sections collected from P7 Chx10TET and TET mice. (C) P4 experimental and control mice were treated with EdU for a brief period (short pulse) to ensure that it would be incorporated into the DNA of only rapidly dividing RPCs. (D, E) The results of measurements of EdU signal intensity (D) and representative confocal images (E) indicate that the experimental (Chx10TET) RPCs divide faster than the control (TET) RPCs (*n* ≥ 5 mice per group).

During retinal development, the population of rapidly dividing early RPCs is replaced by a population of slowly dividing late RPCs, most of which stop dividing and differentiate by P7 [6]. Symmetric division is also characteristic of early RPCs, whereas asymmetric division is characteristic of late RPCs [3, 7]. Given that TET‐deficient RPCs divide predominantly asymmetrically, one might hypothesize that they would cease dividing earlier than control RPCs. However, we found no differences in EdU incorporation into the DNA of Chx10TET versus TET RPCs up to P7 (Figure 2A, Figure S1). The 7th postnatal day (P7) revealed a significant effect of TET enzymes on RPC proliferation in the developing retinas: RPC proliferation can be detected in the center, middle, and in large quantities at the periphery of P7 Chx10TET retinas, while in P7 TET (control) retinas cell proliferation can be partially detected only at the periphery (Figures 1C, 2A,B, Figure S2A,B). Individual dividing RPCs could still be detected throughout the retina on the 10th postnatal day (P10); however, their number was insufficient to establish a difference between the experimental and control groups (Figures 1C, 2A). Thus, contrary to our hypothesis, TET‐deficient RPCs stop dividing not earlier but later than control RPCs. The presence of numerous dividing RPCs in P7 Chx10TET retinas suggests a slower transition of TET‐deficient RPCs from the rapid‐division phase to the slow‐division phase. To test this hypothesis, we used a short pulse (30 min) to incorporate EdU into the DNA of dividing RPCs (Figure 2C). With this approach, only rapidly dividing RPCs would be capable of incorporating EdU into their DNA. In our experiment, we utilized P4 experimental and control mice in which we had previously observed no differences in EdU incorporation when using a standard pulse (Figure 2A). The results of our analysis indicate a high abundance of rapidly dividing RPCs in the center, middle, and periphery of TET‐deficient retinas (Figure 2D,E). Conversely, in the control (TET) retinas, rapidly dividing RPCs were predominantly concentrated in the periphery (Figure 2D,E). Since a high proliferation rate is characteristic of early RPCs and asymmetric division is characteristic of late RPCs, TET‐deficient RPCs exhibit properties of both early and late RPCs. It should be noted that we found no differences in cell death in the developing retinas of Chx10TET versus TET mice (Figure S2C).

### 3.3 TET Enzymes Control RPC Function at the Molecular Level

**FIGURE 3:**
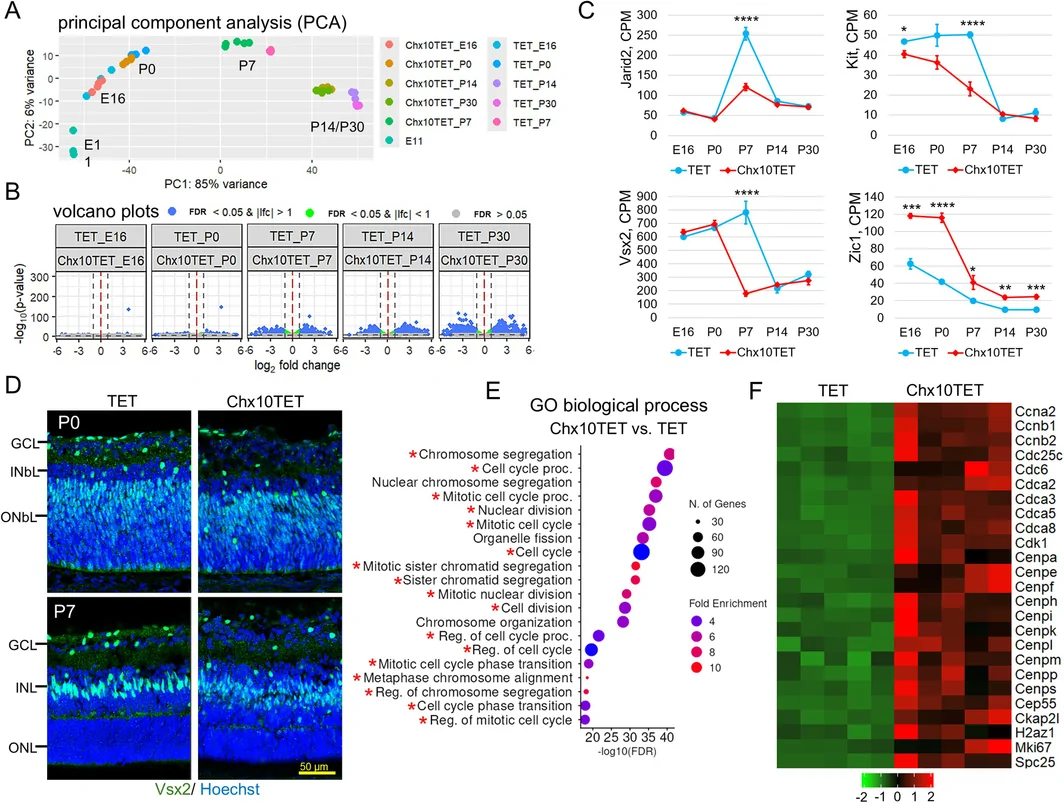
Inactivation of TET enzymes in RPCs significantly affects gene expression during retinal development. (A, B) The principal component analysis (PCA, A), and volcano plots (B) indicate significant differences in gene expression between developing TET‐deficient (Chx10TET) and control (TET) retinas. (C) The expression dynamics of Jarid2, Kit, Zic1, and Vsx2 may reflect RPC dysfunction caused by the inactivation of TET enzymes (****p < 0.0001, ***p < 0.001, **p < 0.01, *p < 0.05). (D) The representative confocal images show differences in Vsx2 expression between TET‐deficient (Chx10TET) and control (TET) retinas on day 0 (P0) and day 7 (P7) of their postnatal development (GCL, ganglion cell layer; INbL, inner neuroblastic layer; INL, inner nuclear layer; ONbL, outer neuroblastic layer; ONL, outer nuclear layer). (E, F) The results of the GO biological process analysis (E, genes with logFC > 1) and the heatmap (F) show increased expression of cell cycle related genes in P7 Chx10TET versus TET retinas.

To elucidate molecular mechanisms responsible for the observed phenotype of TET‐deficient RPCs, we studied gene expression in Chx10TET versus TET retinas using E16, P0, and P7 mice and bulk RNA‐seq. We used P14 and P30 Chx10TET versus TET bulk RNA‐seq data to compare changes occurring in developing and mature retinas [17]. Since the entire mouse retina consists of RPCs on the 11th day of embryonic development (E11), we also used wild‐type E11 retinas to obtain the high‐throughput gene expression profile of RPCs. Our results indicate that the gene expression profiles of E16 and P0 retinas are closer to those of RPCs, whereas the gene expression profiles of P7 retinas are closer to those of mature retinas (P14 and P30; Figure 3A). Differences between the gene expression profiles in Chx10TET versus TET retinas become more distinct with age, reaching a peak at P7 (Figure 3B, Data S2). In mature retinas (P14 and P30), the gene expression profiles differ not by age, but by genotype (Figure 3A,B). We found reduced expression of the Jarid2 and Kit genes in Chx10TET versus TET retinas, both of which are critical for late RPC development (Figure 3C) [27, 28]. There is also a premature decline in the expression of Vsx2 (Chx10), which serves as a marker for RPCs during early retinal development and for bipolar cells during the very late stages of retinal development (Figure 3C,D). It should be noted that the morphology of many cells in P7 TET‐deficient retinas more closely resembles that of RPCs (elongated and showing low Vsx2 expression) than that of bipolar cells (rounded and showing high Vsx2 expression; Figure 3D). At the same time, the expression of Zic1, a transcription factor that sustains the proliferation activities of RPCs, is steadily high in developing TET‐deficient retinas (Figure 3C) [29]. Consistent with our findings of increased RPC proliferation in P7 Chx10TET versus TET retinas, we found increased expression of many genes involved in the cell cycle (Figure 3E,F, Data S2).

### 3.4 TET Enzymes Control the Proper Cellular Composition of the Retina

**FIGURE 4:**
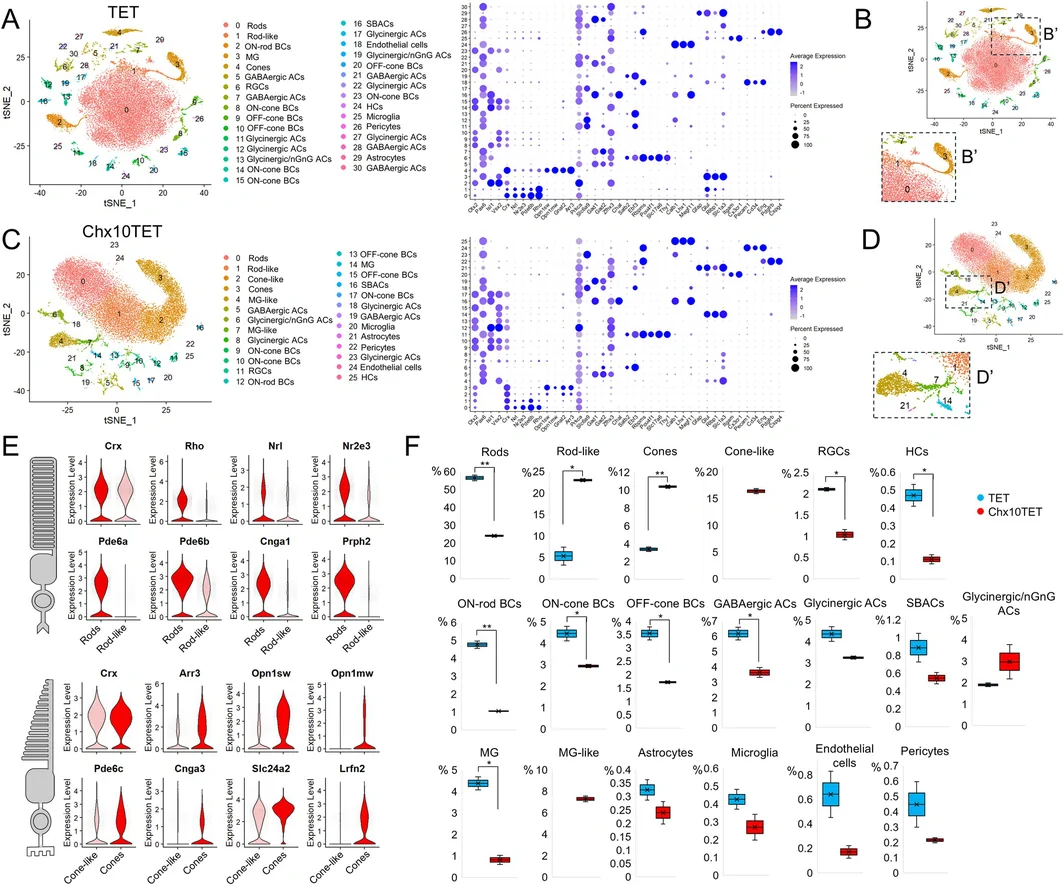
TET‐deficient retinas differ significantly from control retinas in their cellular composition and complexity. (A) Non‐linear dimensional reduction t‐distributed Stochastic Neighbor Embedding (tSNE) technique was used to visualize and explore identified single cell clusters in control (TET) retinas. Each cluster was associated with a specific cell type using known markers. (B) While all clusters correspond to mature cell types in the control retina, cluster 1 likely contains rods that have not yet completed their development. (C–E) The smaller number of clusters (C) and the existence of similar clusters with different levels of marker activity and presence (D, E) indicate less complexity and underdevelopment of TET‐deficient (Chx10TET) retinas compared to control (TET) retinas. (F) The number of cells in each cluster was used to calculate the percentage of the identified cell types in Chx10TET and TET retinas. (*p < 0.05, **p < 0.01).

**FIGURE 5:**
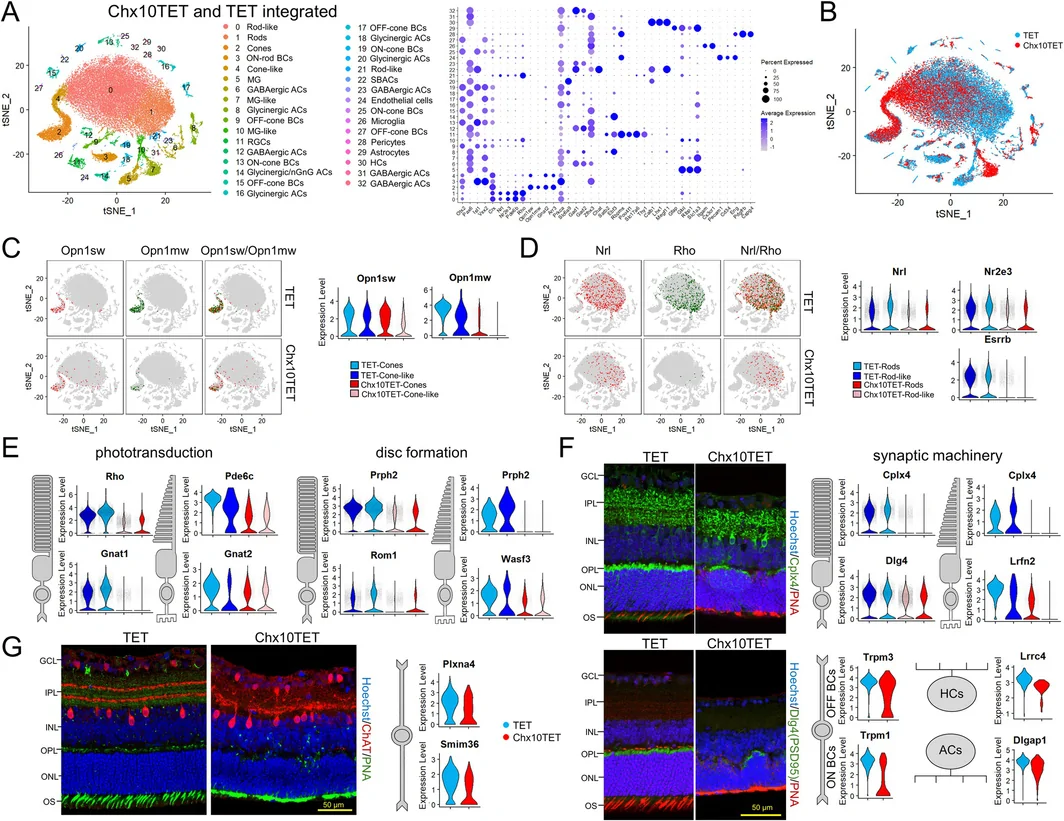
The increase in the number of S‐cones in TET‐deficient retinas occurs at the expense of all retinal cell types. (A) The panel shows the results of the snRNA‐seq data integration. The cell type corresponding to each cluster was determined based on the expression of specific markers. (B) Integration of the snRNA‐seq data indicates that Rod‐like, Cone‐like, and MG‐like cells are predominantly contained in clusters where TET‐deficient cells (Chx10TET) predominate. (C) The peculiarity of TET‐deficient retinas is not only that they have a higher content of Cones and Cone‐like cells, but also that these photoreceptors are mainly S‐Cones and S‐Cone‐like cells. (D) Expression of key genes responsible for rod specification is reduced in cells contained in Chx10TET Rod and Rod‐like clusters. (E, F) Reduced expression of genes responsible for phototransduction (E), outer segment formation (E), and synaptogenesis (F) results in the abnormalities seen in the confocal images (F) of Chx10TET versus TET retinas. (ACs, amacrine cells; BCs, bipolar cells; HCs, horizontal cells) (G) Altered gene expression profiles in TET‐deficient bipolar cells may result in abnormal lamination of the inner plexiform layer (IPL).

Disturbances in the function of TET‐deficient RPCs should be reflected in the structure of the retina. To test the hypothesis, we used single‐nucleus RNA‐seq (snRNA‐seq) and P14 TET and Chx10TET retinas that had just completed their development (at least in the context of normal development). The experiment was repeated two times on TET and Chx10TET retinas using the 10X genomics platform. In each experiment, we obtained gene expression data from more than 10 000 single cell nuclei (more than 50 000 cells in total). The bioinformatics analysis of our snRNA‐seq data obtained on control TET retinas enabled classification of all cells into 31 clusters (Figure 4A). We manually identified which cluster corresponded to which retinal cell type using expression data of known cell markers (Figure 4A, Figure S3). Cluster 1 contained cells expressing rod markers. However, the expression of these markers and the percentage of cells in the cluster 1 that expressed these markers were lower than in the cluster 0 that represents Rods (Figure 4A, Data S3). For this reason, we have designated this cluster as Rod‐like. The position of cluster 1 and the level of expression of rod markers suggest that the cells in it have not yet completed their differentiation into rod photoreceptors (Figure 4B,B’). The same bioinformatics analysis of the snRNA‐seq data obtained on TET‐deficient Chx10TET retinas revealed only 26 clusters (Figure 4C, Figure S5). While the number of clusters identified was smaller in Chx10TET versus TET retinas, we found several clusters with cells expressing Müller glia (MG) markers (Figure 4D). We designated cluster 14 with high expression of these markers as MG, while the remaining two as MG‐like (Figure 4C,D, Data S4). We also detected clusters (1 and 2) whose cells express only photoreceptor (rod or cone) markers but significantly weaker than clusters 0 (Rods) and 3 (Cones). Therefore, we have designated them as Rod‐like and Cone‐like (Figure 4C,E, Data S5). After assigning clusters to retinal cell types, we further determined the percentage of cells in each cluster relative to the total number of cells and compared these percentages between Chx10TET versus TET retinas (Figure 4F). We found that the number of Rods in control retinas significantly exceeds the number of Rods in TET‐deficient retinas (TET 56% ± 2% vs. Chx10TET 24% ± 1%, *p* = 0.003). However, the numbers are opposite for Rod‐like cells (TET 5% ± 2% vs. Chx10TET 22.9% ± 0.3%, *p* = 0.01). The number of Cones in Chx10TET retinas significantly exceeds the number of Cones in control TET retinas (TET 3.4% ± 0.3% vs. Chx10TET 10.4% ± 0.2%, *p* = 0.002). Chx10TET retinas also contain Cone‐like cells (16.4% ± 0.5%), and the sum of the percentages of TET‐deficient Cones and Cone‐like cells is 9 times greater than the percentage of Cones in control TET retinas. Apart from photoreceptors, the percentage of many other types of retinal neurons is lower in TET‐deficient retinas compared to controls (Figure 4F). These findings suggest the underdevelopment and reduced complexity of TET‐deficient retinas. To compare gene expression profiles in Chx10TET versus TET retinas, we integrated our snRNA‐seq data (Figure 5A). We found 33 clusters that included features of Chx10TET and TET retinas (Figures 5A, 4A,C, Figure S5). It should be noted that Rod‐like, Cone‐like and MG‐like clusters contain mostly cells from Chx10TET retinas (Figure 5B). We then estimated the percentage of cells in each cluster relative to the total number of cells, taking into account which retinas they came from (TET vs. Chx10TET): the results turned out to be almost indistinguishable from those obtained before integration (Figure S6). At the same time, our results indicate that in terms of gene expression, TET‐deficient Rods, Rod‐like cells, Cones, and Cone‐like cells are very different from those in control retinas: the expression of many rod and cone genes is significantly reduced in TET‐deficient photoreceptors (Figure 5C–5F, Figure S7, Data S6). The significant decrease in M‐opsin (Opn1mw) expression compared to S‐opsin (Opn1sw) expression in Chx10TET retinas suggests that most Cones and Cone‐like cells in TET‐deficient retinas are S‐Сones or S‐Сone‐like (Figure 5C). We found a significant decrease in the expression of genes responsible for rod specification, rod and cone phototransduction, the formation of outer segments and synaptic terminals (Figure 5D,F, Figure S7, Data S6). This decrease in expression prevents the normal development of outer segments and synaptic terminals in TET‐deficient photoreceptors (Figure 5F). We also found reduced expression of genes responsible for synaptogenesis in TET‐deficient retinal neurons that make up clusters corresponding to bipolar, horizontal, and amacrine cells (BCs, HCs, and ACs; Figure 5F, Figure S7, Data S7). Decreased expression of some genes responsible for lamination in the retina may partly explain the disturbances in the lamination of the IPL in Chx10TET retinas that we observed (cluster 17, Figure 5G).

## 4 Discussion

The retina is a multi‐layered neural tissue located in the eye that is responsible for coding light information into electrical signals that the brain uses to form the sensation of vision [1, 2, 3, 4]. The highly organized structure and cellular composition of the retina are critical to its function, and any disruptions due to impaired development lead to blindness [1, 2, 3, 4, 5]. The development of the retina is determined by the activity of RPCs, whose function is to proliferate and then differentiate into all types of retinal cells. Therefore, it is important to identify genes that are involved in these processes in RPCs. Our findings indicate that the TET gene family is involved in all these processes in RPCs, thereby controlling RPC function in the developing retina.

It is a widely accepted concept that stem cells with unlimited proliferative potential differentiate into progenitors with limited proliferative potential. Functional tissue is formed through the activity of the progenitors. Early progenitors exhibit a high rate of proliferation and symmetric self‐renewal [30, 31, 32]. This leads to an exponential increase in cell number and rapid tissue growth. Early progenitors are also capable of differentiating into all cell types within the tissue directly or indirectly by differentiating into late progenitors, which in turn differentiate into cell types that appear later in development [30, 31, 32]. A defining characteristic of late progenitors is that they divide slowly and asymmetrically, generating one progenitor and one non‐dividing precursor of a certain cell type [30, 31, 32]. Early and late RPCs possess all these properties [3, 6, 7, 8]. In our study, we found that inactivation of TET enzymes in RPCs results in slow retinal growth, a small number of RPCs, and a large number of non‐dividing cells. This phenomenon can be explained by the predominantly asymmetric division of TET‐deficient RPCs, which is characteristic of late RPCs. Such asymmetric division, yielding one dividing RPC and one non‐dividing precursor, would result in a more linear (rather than exponential) growth of the TET‐deficient retina and the subsequent accumulation of non‐dividing cells. At the same time, however, our EdU tests and RNA‐seq data indicate that TET‐deficient RPCs maintained a high rate of proliferation, continuing to divide even during late stages of retinal development, a phase during which cell division could no longer be detected in the control samples. A high rate of proliferation is a characteristic typically associated with early RPCs. Reduced expression of Jarid2 and Kit transcription factors, both of which are critical for late RPC development, and increased expression of Zic1, a transcription factor that supports RPC proliferation, could explain these observations [27, 28, 29]. These findings strongly suggest that TET‐deficient RPCs exhibit characteristics associated with both early and late RPCs. Thus, the TET‐dependent DNA demethylation pathway prevents the mixing of early and late RPC phenotypes and facilitates their manifestation in accordance with the stage of retinal development.

Our results also indicate that TET enzymes control the differentiation of RPCs into various retinal cell types. We observed a high abundance of early‐born S‐cone photoreceptors in TET‐deficient retinas. Conversely, the numbers of other retinal cell types, both early‐born and late‐born, were reduced. This unbalanced distribution of early‐ and late‐born retinal cell types can likely be attributed to the mixed phenotype of TET‐deficient RPCs. However, the influence of the TET family is not limited merely to altering the composition of retinal cell populations; the inactivation of TET enzymes in RPCs impacts the development of all retinal cell types [17, 18, 19]. This effect is particularly pronounced in the development of rod and cone photoreceptors (Figure 5, Figure S7). This phenomenon is explained by the fact that dozens of genes necessary for the development and function of photoreceptors are highly methylated in RPCs and must be demethylated by TET enzymes to be activated in rods and cones [17]. We do not rule out the possibility that many genes essential for the development and function of other retinal cell types are also highly methylated in RPCs. However, identifying these genes requires isolating pure populations of these cell types, which is a challenging task. An even greater challenge is identifying the genes that must undergo demethylation during the transition from early to late RPCs, as separating populations of early and late RPCs is difficult. We consider it possible that, once this challenge is met, the Jarid2 and Kit genes will be found to be highly methylated in early RPCs but have low methylation levels in late RPCs. We also consider the possibility that currently unknown transcription factors inhibiting the expression of Zic1 in late RPCs are highly methylated in early RPCs. This would explain Zic1 increased expression in TET‐deficient retinas.

In conclusion, we have demonstrated that TET enzymes play an important role in RPC function. TET family maintains the phenotype of early and late RPCs and further promotes RPC differentiation into various retinal cell types; most notably rods and cones. Further research is needed to identify RPC genes whose timely demethylation by TET enzymes is required for RPCs to function normally in the developing retina.

## Author Contributions

Dmitry Ivanov conceived and supervised the project. Galina Dvoriantchikova, Michelle Fleishaker, and Dmitry Ivanov performed the experiments and the data analyses. Galina Dvoriantchikova and Dmitry Ivanov assisted with the bioinformatic analysis. Galina Dvoriantchikova, Michelle Fleishaker, Byron L. Lam, and Dmitry Ivanov assisted with the research design, data interpretation, manuscript writing, and editing. All authors have read and agreed to the published version of the manuscript.

## Funding

This work was supported by HHS | NIH | National Eye Institute (NEI), R01 EY035235, P30 EY014801. Research to Prevent Blindness (RPB), GR027195.

## Conflicts of Interest

The authors declare no conflicts of interest.

## Supporting information




**Figure S1:** Mean fluorescent EdU signal intensity and the thickness of the retinal layers were measured every 200 μm from the head of the optic nerve. (A) E16, (B) P0, (C) P4, (D) P7, (E) P10 (*n* ≥ 5 mice per group, per age; ****p < 0.0001, ***p < 0.01, *p < 0.05; INbL—inner neuroblastic layer, ONbL—outer neuroblastic layer, GCL—ganglion cell layer, INL—inner nuclear layer, ONL—outer nuclear layer, IPL—inner plexiform layer, OPL—outer plexiform layer).

**Figure S2:** TET enzymes affect cell proliferation in developing retinas. (A) Low EdU incorporation into DNA indicates virtually no cell proliferation in P7 control TET retinas. (B) High EdU incorporation into DNA indicates high cell proliferation rate in TET‐deficient Chx10TET retinas. (C) We did not find TUNEL‐positive dead cells in P4 Chx10TET and TET retinas. Positive controls were treated with DNase I to induce DNA strand breaks (red—TUNEL‐positive dead cells, blue—Hoechst 33342‐positive cell nuclei).

**Figure S3:** Each cluster identified by analysis of snRNA‐seq data from control (TET) retinas was assigned a specific cell type/subtype based on known markers. Eight markers were particularly helpful in identifying retinal cell types: Otx2, which is a marker of cone, rod, and bipolar cells (BCs); Pax6—a marker of amacrine cells (ACs), horizontal cells (HCs), retinal ganglion cells (RGCs), and Muller glia (MG); Isl1—a marker of ON BCs, RGCs, and starburst amacrine cells (SBACs); Vsx2—a marker of BCs and MG; Crx—a marker of cone and rod photoreceptors, Gad1 and Gad2—markers of GABAergic ACs, and Slc6a9—a marker of glycinergic ACs.

**Figure S4:** Each cluster identified by analysis of snRNA‐seq data from TET‐deficient (Chx10TET) retinas was assigned a specific cell type/subtype based on known markers.

**Figure S5:** Integration of the snRNA‐seq TET and Chx10TET data was performed with subsequent clustering using R toolkit Seurat. Each cluster identified was assigned a specific cell type/subtype based on known markers.

**Figure S6:** The percentage of TET‐deficient (Chx10TET) and control (TET) cells was determined for each cluster after data integration. (*p < 0.05, **p < 0.01).

**Figure S7:** Several cell types, most notably rods and cones, are underdeveloped in TET‐deficient retinas. SnRNA‐seq data integration allowed comparison of gene expression within clusters of Chx10TET and TET retinas.

**Data S1:** The R code that was used in snRNA‐seq analysis.

**Data S2:** The edgeR Bioconductor package was applied to perform the differential gene expression analysis using bulk RNA‐seq data collected from E11, E16, P0, P7, P14, and P30 TET‐deficient.

**Data S3:** R toolkit Seurat was used to calculate expression levels and to compare these expression levels pairwise between clusters 0, and 1. SnRNA‐seq data obtained from P14 control (TET).

**Data S4:** R toolkit Seurat was used to calculate expression levels and to compare these expression levels pairwise between clusters 4, 7, and 14 (MG‐like cells and MG). SnRNA‐seq data obtained.

**Data S5:** R toolkit Seurat was used to calculate expression levels and to compare these expression levels pairwise between rod‐related (0 and 1) and cone‐related (2 and 3) clusters. SnRNA‐seq data.

**Data S6:** Integration of the snRNA‐seq data using R toolkit Seurat allowed comparison of gene expression between and within clusters (0, 1, 2, and 4) of TET‐deficient (Chx10TET) and control (TET).

**Data S7:** Integration of the snRNA‐seq data using R toolkit Seurat allowed comparison of gene expression within bipolar cell (BC)‐related (13, 15, and 17), amacrine cell (AC)‐related (6, 12, 16, and).

## Data Availability

The datasets collected and analyzed in the study are available in the BioProject database (accession numbers PRJNA1286813 and PRJNA1121391) and in the article/Supporting Information.
