# Supplementary material for: Ten‐Eleven Translocation Enzymes Control the Rate and Mode of Retinal Progenitor Cell Division in the Developing Retina
Source: FASEB J. 2026 Sep 25;40(19):e72287. doi: 10.1096/fj.202603335R (PMC13614436; doi:10.1096/fj.202603335R)
Supplement: Supplementary file 1 [file FSB2-40-e72287-s001.pdf]

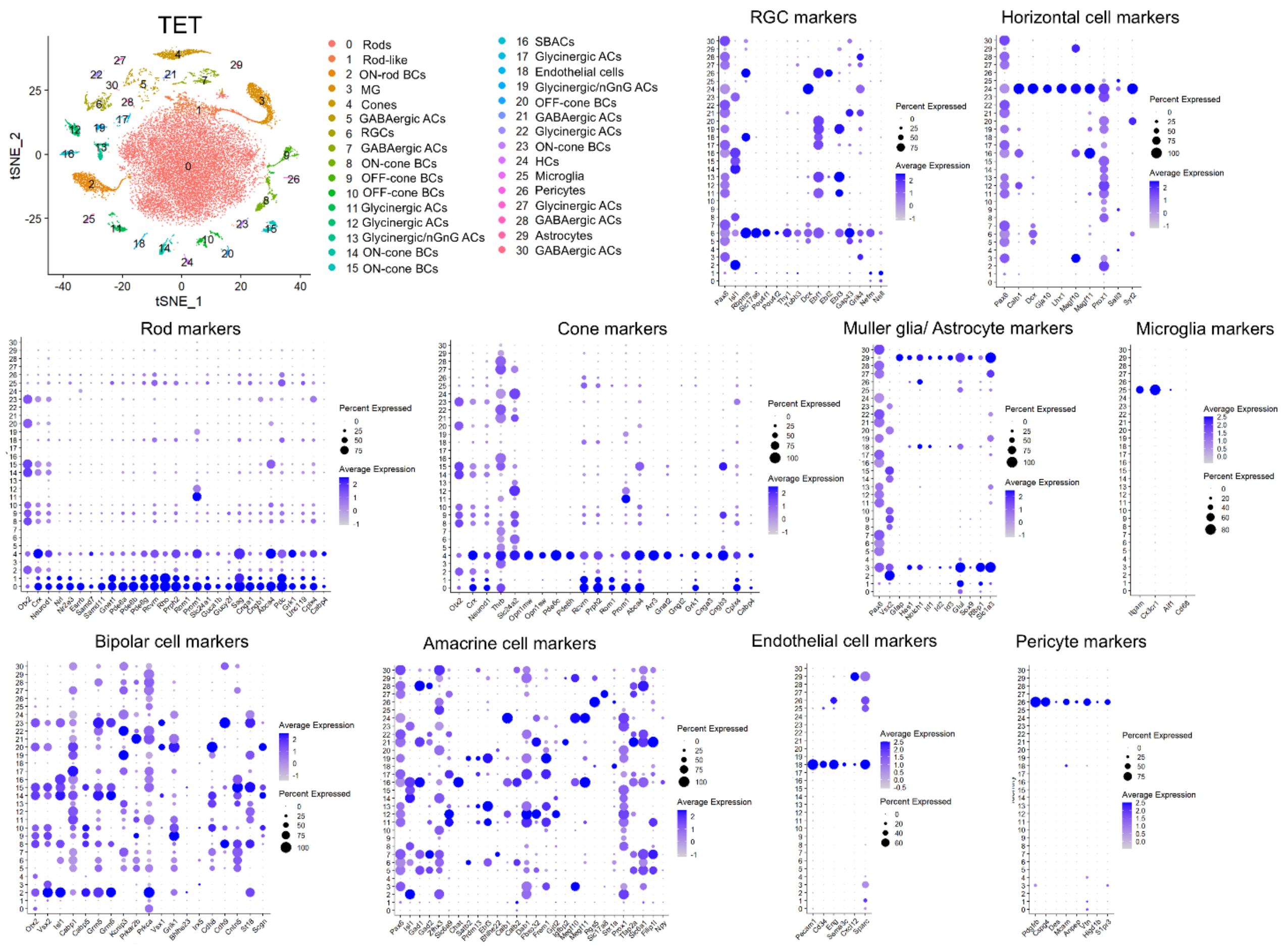

Supplementary Figure S3. Each cluster identified by analysis of snRNA-seq data from control (TET) retinas was assigned a specific cell type/subtype based on known markers. Eight markers were particularly helpful in identifying retinal cell types: Otx2, which is a marker of cone, rod, and bipolar cells (BCs); Pax6 - a marker of amacrine cells (ACs), horizontal cells (HCs), retinal ganglion cells (RGCs), and Muller glia (MG); Isl1 – a marker of ON BCs, RGCs, and starburst amacrine cells (SBACs); Vsx2 – a marker of BCs and MG; Crx – a marker of cone and rod photoreceptors, Gad1 and Gad2 – markers of GABAergic ACs, and Slc6a9 – a marker of glycinergic ACs.
