# Supplementary material for: Ten‐Eleven Translocation Enzymes Control the Rate and Mode of Retinal Progenitor Cell Division in the Developing Retina
Source: FASEB J. 2026 Sep 25;40(19):e72287. doi: 10.1096/fj.202603335R (PMC13614436; doi:10.1096/fj.202603335R)
Supplement: Supplementary file 2 [file FSB2-40-e72287-s003.pdf]

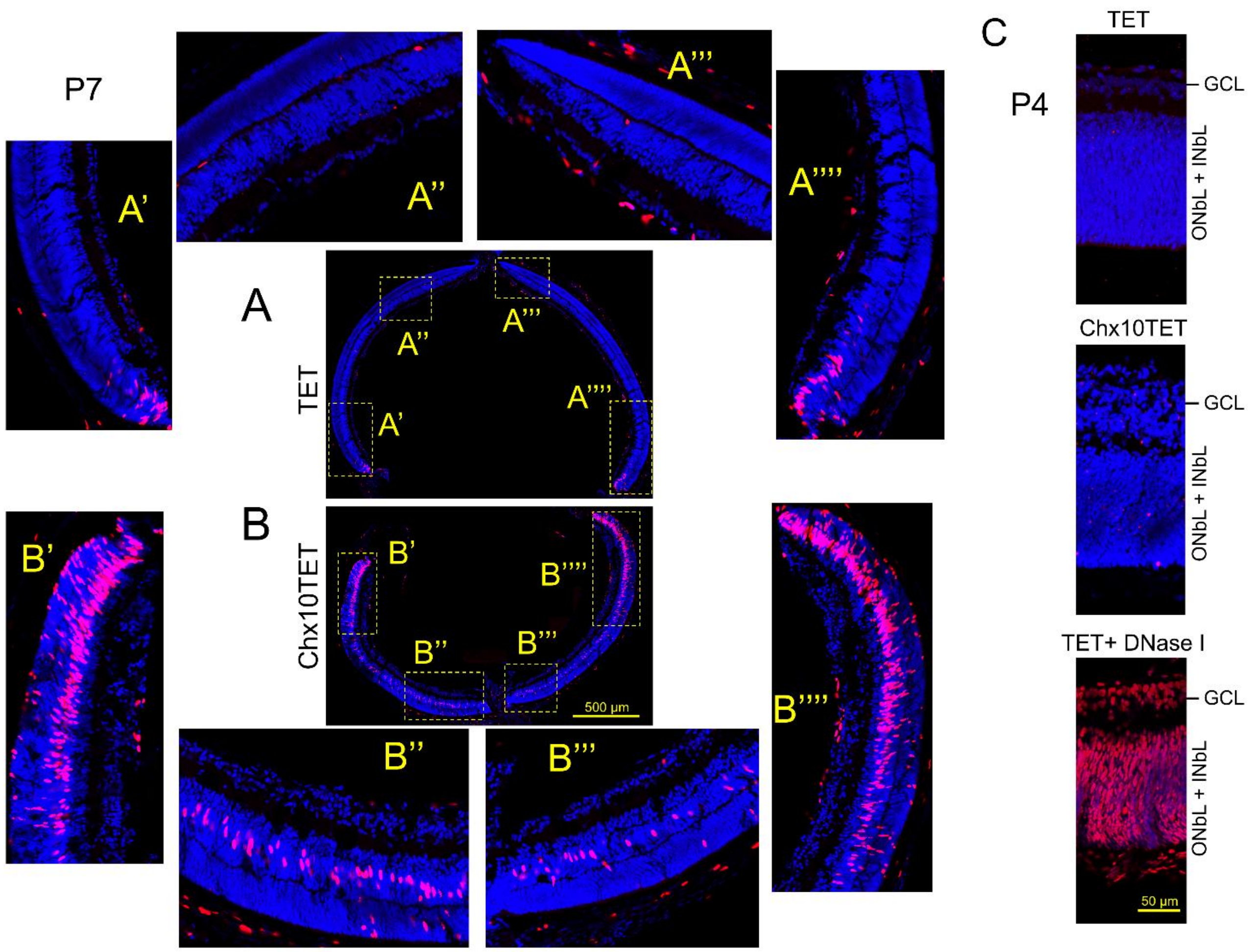

Supplementary Figure S2. TET enzymes affect cell proliferation in developing retinas. **A)** Low EdU incorporation into DNA indicates virtually no cell proliferation in P7 control TET retinas. **B)** High EdU incorporation into DNA indicates high cell proliferation rate in TET-deficient Chx10TET retinas. **C)** We did not find TUNEL-positive dead cells in P4 Chx10TET and TET retinas. Positive controls were treated with DNase I to induce DNA strand breaks (red - TUNEL-positive dead cells, blue - Hoechst 33342-positive cell nuclei).
