# Supplementary material for: Ten‐Eleven Translocation Enzymes Control the Rate and Mode of Retinal Progenitor Cell Division in the Developing Retina
Source: FASEB J. 2026 Sep 25;40(19):e72287. doi: 10.1096/fj.202603335R (PMC13614436; doi:10.1096/fj.202603335R)
Supplement: Supplementary file 3 [file FSB2-40-e72287-s005.pdf]

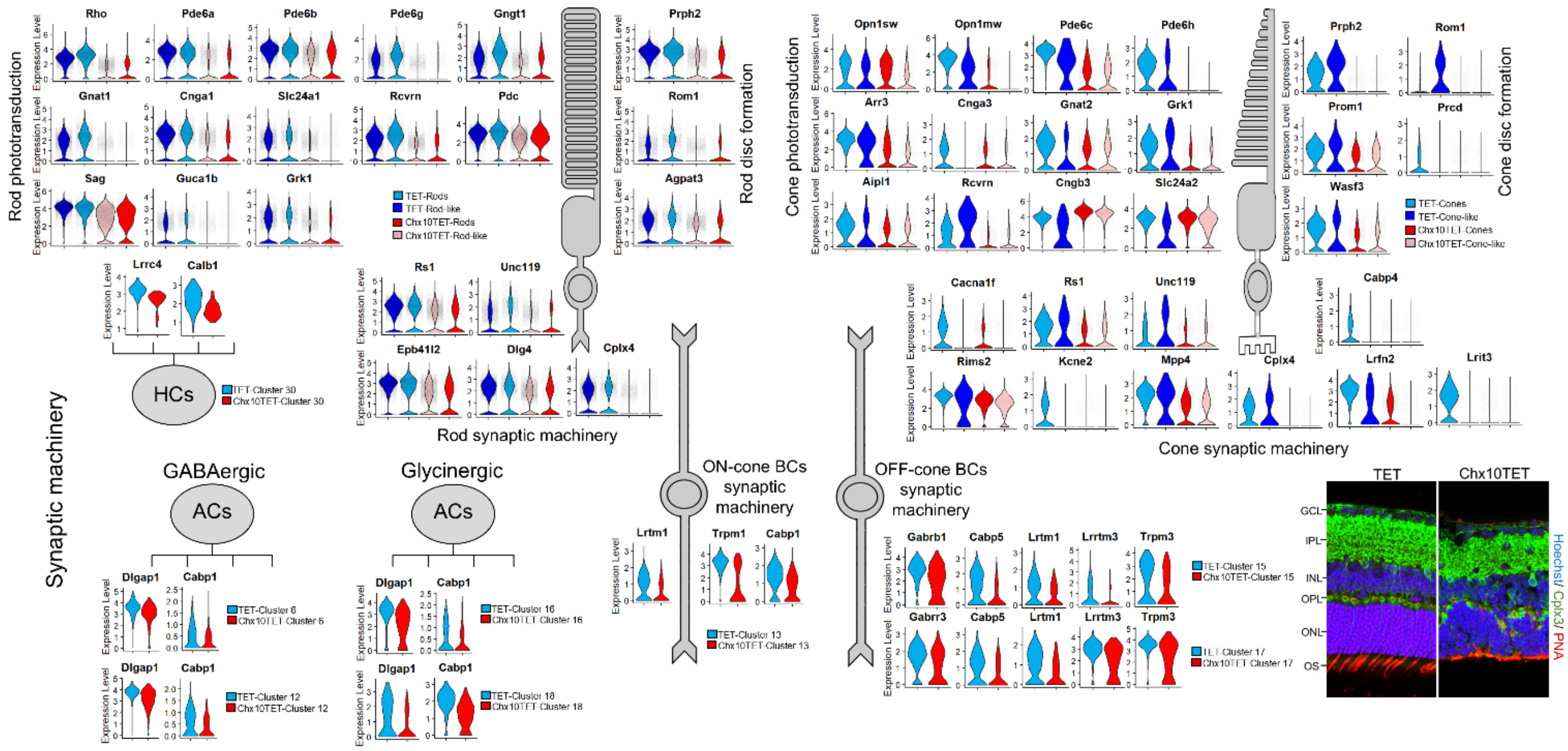

Supplementary Figure S7. Several cell types, most notably rods and cones, are underdeveloped in TET-deficient retinas. SnRNA-seq data integration allowed comparison of gene expression within clusters of Chx10TET and TET retinas.
