# Supplementary material for: Ten‐Eleven Translocation Enzymes Control the Rate and Mode of Retinal Progenitor Cell Division in the Developing Retina
Source: FASEB J. 2026 Sep 25;40(19):e72287. doi: 10.1096/fj.202603335R (PMC13614436; doi:10.1096/fj.202603335R)
Supplement: Supplementary file 4 [file FSB2-40-e72287-s006.pdf]

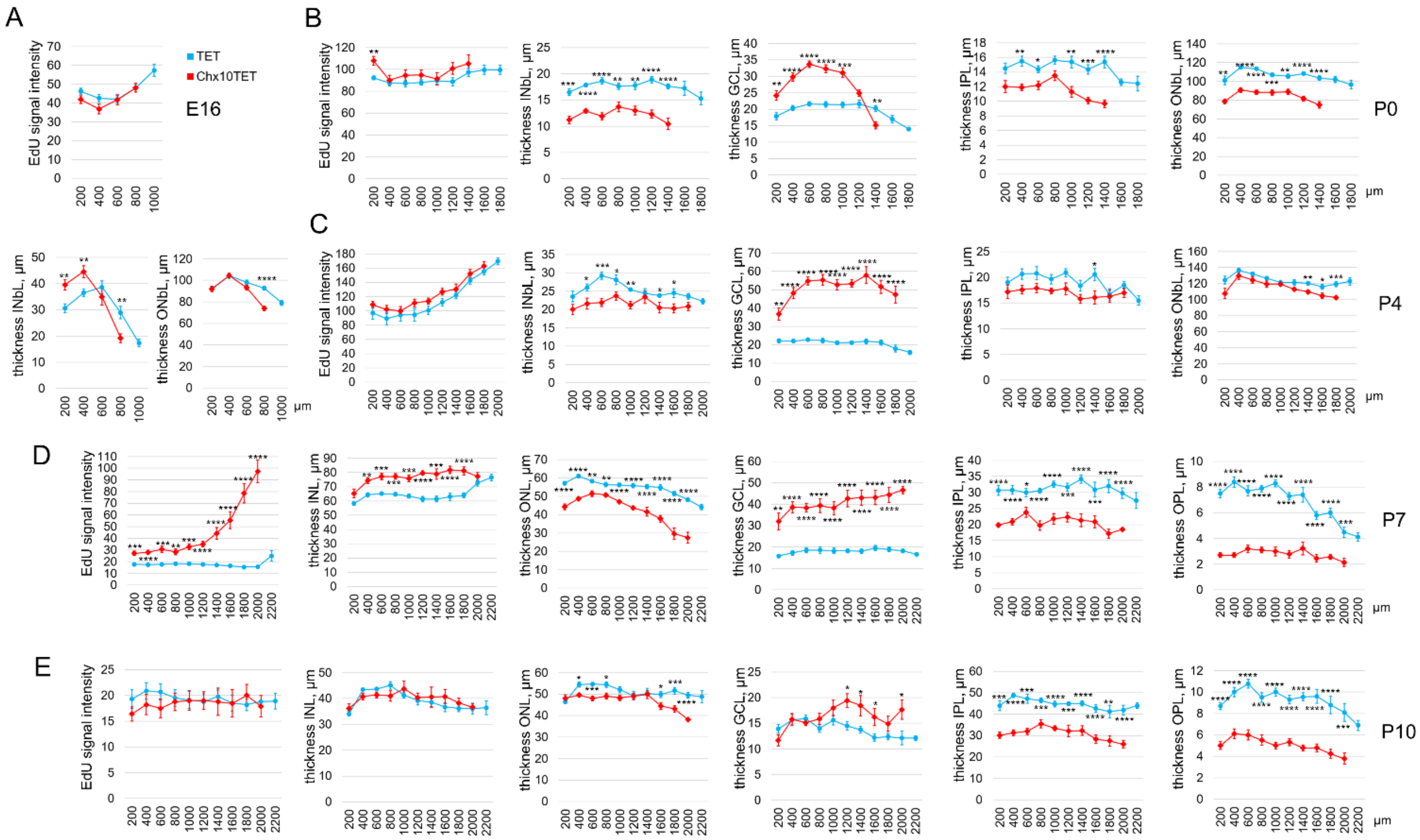

Supplementary Figure S1. Mean fluorescent EdU signal intensity and the thickness of the retinal layers were measured every 200 µm from the head of the optic nerve. **A)** E16, **B)** P0, **C)** P4, **D)** P7, **E)** P10 (n ≥ 5 mice per group, per age; ****P value < 0.0001, ***P value <0.001, **P value < 0.01, *P value < 0.05; INbL - inner neuroblastic layer, ONbL - outer neuroblastic layer, GCL – ganglion cell layer, INL – inner nuclear layer, ONL – outer nuclear layer, IPL – inner plexiform layer, OPL – outer plexiform layer).
