# Supplementary material for: Ten‐Eleven Translocation Enzymes Control the Rate and Mode of Retinal Progenitor Cell Division in the Developing Retina
Source: FASEB J. 2026 Sep 25;40(19):e72287. doi: 10.1096/fj.202603335R (PMC13614436; doi:10.1096/fj.202603335R)
Supplement: Supplementary file 5 [file FSB2-40-e72287-s007.pdf]

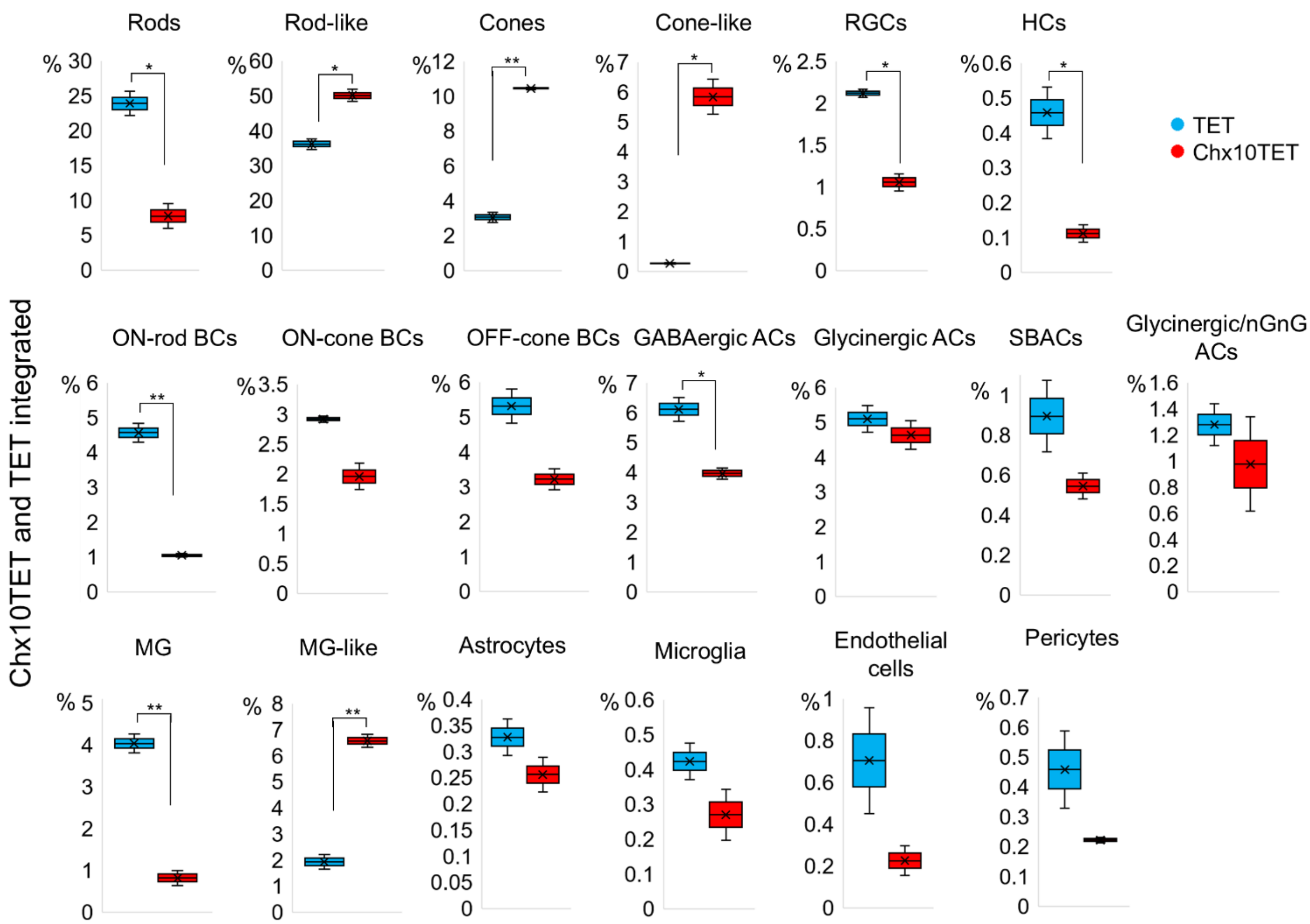

Supplementary Figure S6. The percentage of TET-deficient (Chx10TET) and control (TET) cells was determined for each cluster after data integration. (* - P-value < 0.05, ** - P-value < 0.01)
