# Supplementary material for: Ten‐Eleven Translocation Enzymes Control the Rate and Mode of Retinal Progenitor Cell Division in the Developing Retina
Source: FASEB J. 2026 Sep 25;40(19):e72287. doi: 10.1096/fj.202603335R (PMC13614436; doi:10.1096/fj.202603335R)
Supplement: Supplementary file 6 [file FSB2-40-e72287-s008.pdf]

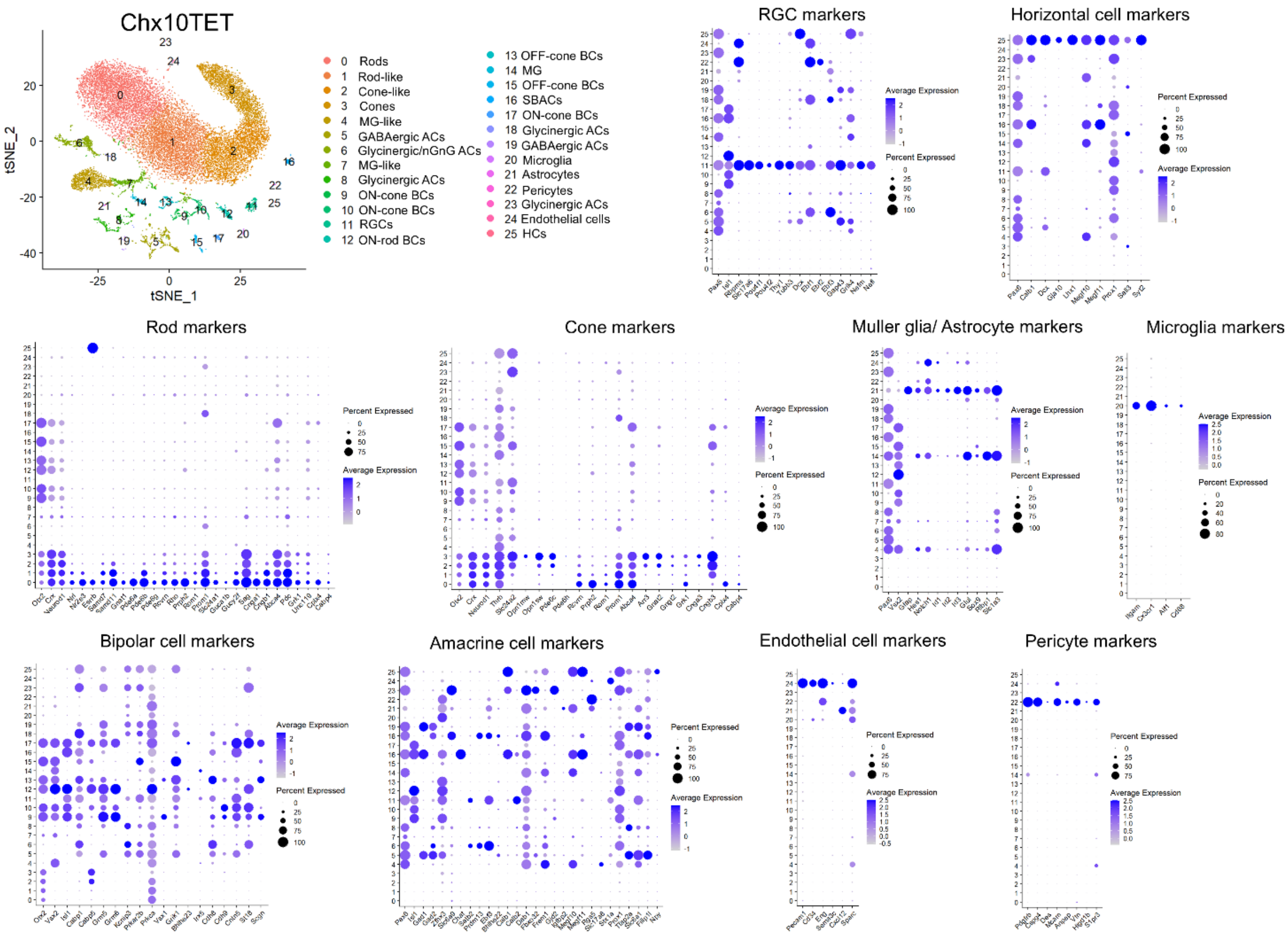

Supplementary Figure S4. Each cluster identified by analysis of snRNA-seq data from TET-deficient (Chx10TET) retinas was assigned a specific cell type/subtype based on known markers.
