# Supplementary material for: Ten‐Eleven Translocation Enzymes Control the Rate and Mode of Retinal Progenitor Cell Division in the Developing Retina
Source: FASEB J. 2026 Sep 25;40(19):e72287. doi: 10.1096/fj.202603335R (PMC13614436; doi:10.1096/fj.202603335R)
Supplement: Supplementary file 7 [file FSB2-40-e72287-s010.pdf]

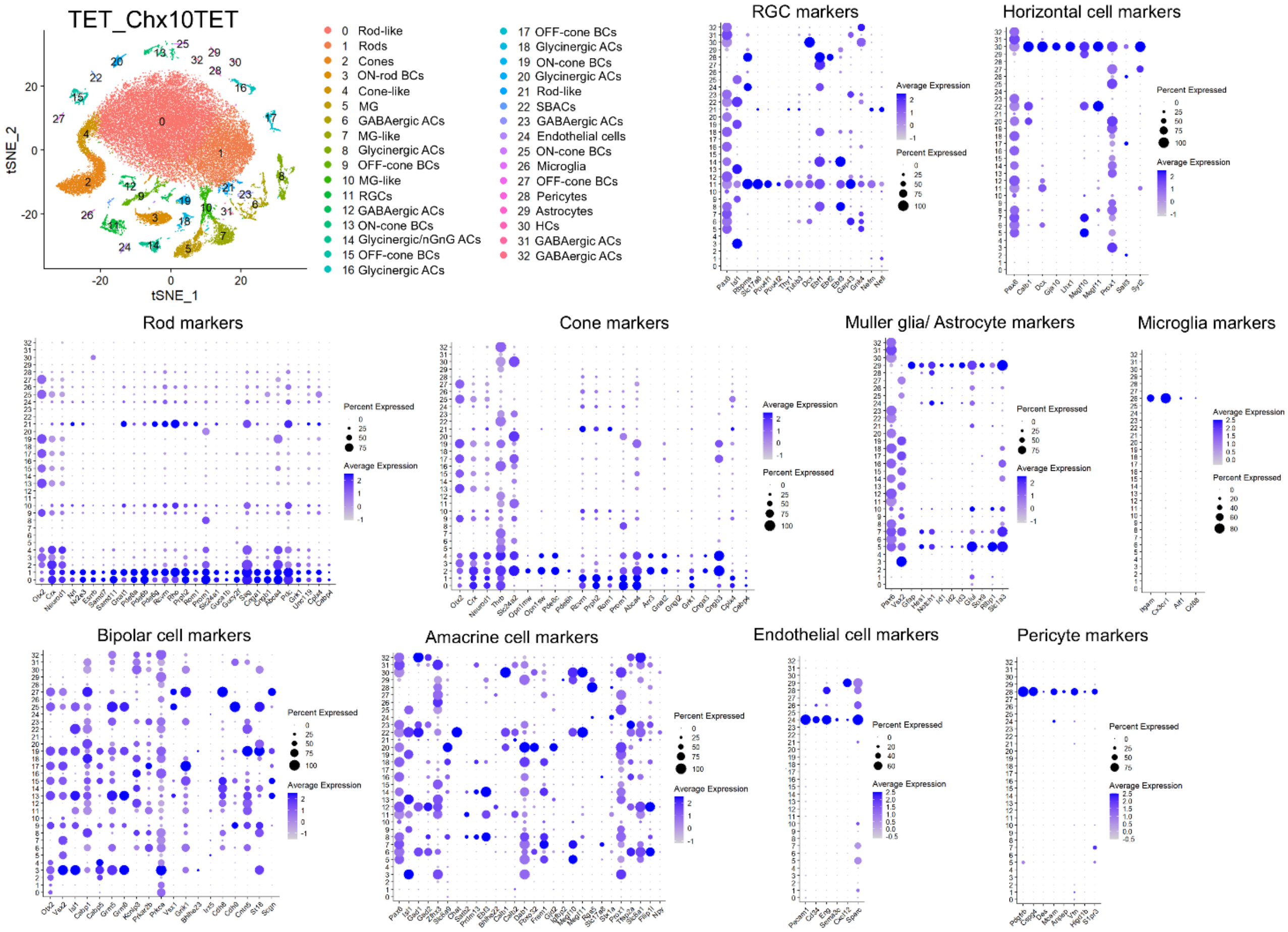

Supplementary Figure S5. Integration of the snRNA-seq TET and Chx10TET data was performed with subsequent clustering using R toolkit Seurat. Each cluster identified was assigned a specific cell type/subtype based on known markers.
